# Genome-wide identification, phylogeny, and expression analysis of GRF transcription factors in pineapple (*Ananas comosus*)

**DOI:** 10.3389/fpls.2023.1159223

**Published:** 2023-04-14

**Authors:** Wen Yi, Aiping Luan, Chaoyang Liu, Jing Wu, Wei Zhang, Ziqin Zhong, Zhengpeng Wang, Mingzhe Yang, Chengjie Chen, Yehua He

**Affiliations:** ^1^ Key Laboratory of Biology and Germplasm Enhancement of Horticultural Crops in South China, Ministry of Agriculture and Rural Areas, College of Horticulture, South China Agricultural University, Guangzhou, China; ^2^ Tropical Crops Genetic Resources Institute, Chinese Academy of Tropical Agricultural Sciences, Haikou, China

**Keywords:** pineapple, GRF gene family, phylogeny analysis, flower organ, expression analysis

## Abstract

**Background:**

Pineapple is the only commercially grown fruit crop in the Bromeliaceae family and has significant agricultural, industrial, economic, and ornamental value. GRF (growth-regulating factor) proteins are important transcription factors that have evolved in seed plants (embryophytes). They contain two conserved domains, QLQ (Gln, Leu, Gln) and WRC (Trp, Arg, Cys), and regulate multiple aspects of plant growth and stress response, including floral organ development, leaf growth, and hormone responses. The GRF family has been characterized in a number of plant species, but little is known about this family in pineapple and other bromeliads.

**Main discoveries:**

We identified eight GRF transcription factor genes in pineapple, and phylogenetic analysis placed them into five subfamilies (I, III, IV, V, VI). Segmental duplication appeared to be the major contributor to expansion of the *AcGRF* family, and the family has undergone strong purifying selection during evolution. Relative to that of other gene families, the gene structure of the GRF family showed less conservation. Analysis of promoter *cis*-elements suggested that *AcGRF* genes are widely involved in plant growth and development. Transcriptome data and qRT-PCR results showed that, with the exception of *AcGRF5*, the *AcGRF*s were preferentially expressed in the early stage of floral organ development and *AcGRF2* was strongly expressed in ovules. Gibberellin treatment significantly induced *AcGRF7/8* expression, suggesting that these two genes may be involved in the molecular regulatory pathway by which gibberellin promotes pineapple fruit expansion.

**Conclusion:**

AcGRF proteins appear to play a role in the regulation of floral organ development and the response to gibberellin. The information reported here provides a foundation for further study of the functions of *AcGRF* genes and the traits they regulate.

## 1 Introduction

Pineapple is one of the three most widely cultivated tropical fruit crops in the world and the only commercially grown member of the Bromeliaceae family (Lobo and Paull, 2017). Because of its excellent flavor and texture, it has been called the queen of fruits (Baruwa, 2013). Its unique shape, fiber content, and nutritional value give it an important place in medicine and industry (Sanewski et al., 2018). However, adverse environmental factors such as temperature extremes, as well as changes in hormone levels, can seriously affect pineapple growth and development, reducing yield and quality. Exploring the mechanisms that regulate flower and fruit development and stress responses is important for maintaining the commercial value of pineapple.

Transcription factors regulate the ability of cells to express different genes and thereby control development (Riechmann et al., 2000; Levine and Tjian, 2003). Growth-regulating factor (GRF) proteins are widespread transcription factors in plants, and their highly conserved N-terminal QLQ and WRC domains are their most prominent feature (Van der Knaap et al., 2000). These two domains have different functions; the QLQ domain is involved in protein interaction and is rich in aromatic/hydrophobic amino acids (Kim and Kende, 2004), whereas the WRC domain mediates DNA-binding ability (Osnato et al., 2010; Kim et al., 2012; Kuijt et al., 2014).

Since the first GRF protein was discovered in rice (*Oryza sativa*) (Van der Knaap et al., 2000; Omidbakhshfard et al., 2015), GRFs have been shown to influence almost all plant growth and developmental processes, including leaf growth (Kim and Kende, 2004; Kim and Lee, 2006; Arvidsson et al., 2011; Debernardi et al., 2014; Wu et al., 2014; Zhang et al., 2021), floral organ development (Arvidsson et al., 2011; Pu et al., 2012; Liang et al., 2013; Liu H. et al., 2014; Pajoro et al., 2014; Lee et al., 2017), root development (Bao et al., 2014), seed oil content (Bao et al., 2014), plant lifespan, and stress responses (Kim et al., 2012; Casadevall et al., 2013; Liu J. et al., 2014). Understanding the roles of GRFs is therefore important for plant growth research and genetic improvement.

Recent studies have shown that *AtGRF*s from the model plant *Arabidopsis thaliana* can significantly improve the transgenic transformation efficiency of a variety of crops, in addition to performing some of the functions above. For example, overexpression of *AtGRF5* and its orthologs increases transformation efficiency, callus cell proliferation, and transgenic bud formation in *Beta vulgaris*, corn (*Zea mays*), soybean (*Glycine max*), and *Helianthus annuus* (Debernardi et al., 2020; Kong et al., 2020). Expression of a GRF4–GIF1 fusion protein significantly increased the regeneration efficiency, regeneration rate, and somatic embryogenesis of wheat (*Triticum aestivum*) and rice (*Oryza sativa*) (Debernardi et al., 2020). Although there are multiple literature reports on the functions of GRF family members, detailed genome-wide phylogenetic and functional studies of GRF genes are not yet available for pineapple or other bromeliads.

To better understand the evolutionary dynamics of GRF genes in pineapple and explore their potential regulatory roles in flower and fruit development and hormone and stress responses, we identified eight AcGRF genes in pineapple and performed a series of analyses, documenting their chromosome locations, motif compositions, evolutionary relationships, genomic collinearity, and selection pressure. We also investigated the potential roles of these *AcGRF*s using interaction network prediction and gene expression analysis. These results provide insights into the functions of GRF family members in pineapple growth and development.

## 2 Materials and methods

### 2.1 Data sources and sequence retrieval

All protein sequences of pineapple were obtained from the Pineapple Genome Project (Ming et al., 2015). Sequences of nine *A. thaliana* GRF genes and 12 rice GRF genes were obtained from previous studies (Kim et al., 2003; Choi et al., 2004). The corresponding protein sequences were downloaded from the *Arabidopsis* Information Resource Library (TAIR) (http://www.arabidopsis.org/) and the UniProt Database (https://www.uniprot.org) (Boutet et al., 2016; Cheng et al., 2017). Protein sequences from *Phalaenopsis equestris* and *Nymphaea colorata* were obtained from recent studies (Zhang et al., 2017; Zhang and Chen, 2019), and the *Amborella trichopoda* proteins were downloaded from the PLAZA database (https://bioinformatics.psb.ugent.be/plaza/) (Van Bel et al., 2017). Protein sequences for *Vitis vinifera*, *Sorghum bicolor*, *Musa acuminata*, and other species were obtained from Phytozome (http://www.phytozome.net/).

### 2.2 Identification and classification of *GRFs*

We used two strategies to identify *GRF* genes in pineapple. First, we performed a local BlastP search of pineapple protein sequences using GRF protein sequences from *A. thaliana* as queries. We then obtained hidden Markov models of the QLQ (PF08880) and WRC (PF08879) domains from the Pfam database (http://pfam.xfam.org/) and used them to query the pineapple protein files using TBtools with an E value of 1e−10 (Chen C. et al., 2020). We identified 21 protein sequences as candidate GRFs in pineapple. We confirmed the presence of GRF core sequences using the Batch CD-Search and SMART programs, further examining all candidate genes that appeared to contain QLQ and WRC domains in the BlastP and HMMER search results. Each candidate gene was then manually checked, and the structurally annotated gene was corrected to ensure that there were conserved heptapeptide sequences at the N terminus of the predicted QLQ and WRC domains. Thirteen protein sequences with no or incomplete QLQ or WRC domains were removed. We identified GRF genes in 23 additional species using the same method, and the complete set of GRF protein sequences was used to study their evolutionary relationships. The ExPASy ProtParam database (https://web.expasy.org/protparam/) was used to analyze the physicochemical properties of the AcGRF genes/proteins, including coding region length, number of amino acids, molecular weight (MW), and theoretical isoelectric point (pI).

The subcellular localizations of the AcGRFs were predicted using Plant-mPLoc (http://www.csbio.sjtu.edu.cn/bioinf/plant-multi/#), the transmembrane domains using TMHMM (http://www.cbs.dtu.dk/services/TMHMM/), and the signal peptides using SignalP (http://www.cbs.dtu.dk/services/SignalP/).

We next characterized the evolutionary relationships among *GRF* genes from various species and assigned the putative pineapple *GRF* genes to specific subfamilies. Multiple-sequence alignments of amino acid sequences were constructed using MAFFT with default parameters, and a species-tree of 26 taxons was constructed using their protein sequences with the maximum likelihood method and 1,000 bootstrap replicates in OrthoFinder with the following parameters: orthofinder -f dataset -M msa -S diamond -t fasttree -t 16 -a 16.

### 2.3 Chromosome locations, gene structures, and conserved motifs of the AcGRFs

We obtained chromosome locations and genetic structures of each *AcGRF* gene from the pineapple genome annotation file (Ming et al., 2015). The data were then integrated and plotted using TBtools (Chen C. et al., 2020). We identified conserved motifs shared among the AcGRF proteins (Bailey et al., 2009) using MEME tools with the following parameters: maximum number of cardinal orders, 10; minimum width, 20; maximum width, 50.

### 2.4 Duplication, collinearity, and evolutionary analysis of the *AcGRF* gene family

We performed collinearity analysis using the method described in TBtools (Chen et al., 2022). We first prepared the pineapple genome and annotation file, then used it as input for collinearity analysis using the “One Step MCScanX Wrapper” function with the following parameters: CPUs for BlastP, 8; e-value, 1e−3; number of blast hits, 10. Dispersed, proximal, tandem, and segmental/WGD duplicates in the *AcGRF* family were identified using TBtools. We connected segmentally duplicated gene pairs by red and green arcs in the Circos plot. We also performed collinearity analysis of GRF genes in pineapple and other plant species, including *A. thaliana*, *V. vinifera*, *A. trichopoda*, *N. colorata*, *O. sativa*, *S. bicolor*, *M. acuminata*, *Phalaenopsis equestris*, and *Spirodela polyrhiza*. TBtools was used to visualize a portion of the results.

### 2.5 Identification of *cis*-elements in the *AcGRF* promoters

The 2,000-bp sequence upstream of each *AcGRF* gene was extracted using TBtools and defined as the promoter region. PlantCARE software (http://bioinformatics.psb.ugent.be/webtools/plantcare/html/) was then used to predict the *cis*-elements in the promoter region (Lescot, 2002), and the results were visualized using TBtools.

### 2.6 Expression profiles of *AcGRF* genes in different pineapple tissues

Earlier transcriptome studies have generated data on *AcGRF* gene expression in floral organs at different developmental stages (Wang et al., 2020) and in various fruit tissues during development (Mao et al., 2018). The transcriptome data from both projects were downloaded from NCBI using the project IDs PRJEB38680 and PRJNA483249. The two datasets were analyzed separately using FPKM values. TBtools was used to transform the FPKM values to log_2_(FPKM+1) values and generate an expression heatmap for the relevant genes.

### 2.7 Hormone and stress treatments, RNA extraction, and RT-PCR

Well-grown “Shenwan” calli were sampled after exposure to 0.1 mM jasmonic acid (JA), 0.1 mM abscisic acid (ABA), 0.1 mM auxin (IAA), 0.1 mM gibberellin (GA), or 150 mM NaCl in suspension culture medium for 0, 4, 8, 16, 24, 36, and 48 h. All materials were immediately frozen in liquid nitrogen and stored at −80°C for subsequent RNA extraction.

The CWBIO RNApure Plant Kit (DNase I) was used to extract total RNA. RNA quality was checked by agarose gel electrophoresis, and RNA concentration was estimated using a Nanodrop ND-1000 spectrophotometer. First-strand cDNA was synthesized from DNA-free RNA using the HiScript II First-Strand cDNA Synthesis Kit (YEASEN) according to the manufacturer’s protocol. qRT-PCR was performed on a Roche LightCycler 480 instrument with SYBR Green Master Mix (YEASEN). The total reaction volume was 10 μL and contained 5 μL SYBR mix, 0.4 μL upstream and downstream primer mix (2.5 μM), 0.5 μL cDNA template, and 4.1 μL ddH_2_O. Each reaction was performed in triplicate. The reaction conditions were 95°C for 30 s, followed by 40 cycles of 95°C for 10 s and 60°C for 30 s. Relative gene expression was calculated by the 2^−ΔΔCt^ method with β-actin as the internal reference gene. Primer sequences used in this study are provided in **Table S10**.

### 2.8 Protein–protein interaction network prediction

All AcGRF protein sequences were submitted to the STRING website (http://string-db.org), and their rice orthologs were selected as a reference. After completing the BLAST step, the network was built using the proteins with the highest scores (bitscores). Proteins that were not predicted to interact with any other proteins were removed.

### 2.9 miRNA target prediction for *AcGRFs*

Pineapple miRNAs were obtained from previous studies (Yusuf et al., 2015), and *AcGRF* genes targeted by miRNAs were predicted using psRNATarget (https://www.zhaolab.org/psRNATarget/) with default parameters while selecting target accessibility as described in Dai et al. (2018). We visualized the interactions among the predicted miRNAs and the corresponding target *AcGRF* genes using TBtools v1.106.

## 3 Results and analysis

### 3.1 Physicochemical properties of AcGRF transcription factors

We identified eight *GRF* gene family members with intact QLQ and WRC domains in pineapple and named them *AcGRF1* to *AcGRF8* based on their chromosome locations (**Table 1**). The eight *GRFs* encoded proteins with 249 (AcGRF4) to 612 (AcGRF6) amino acids, molecular weights (MWs) from 25.1 kDa (AcGRF4) to 65.7 kDa (AcGRF7), and isoelectric points (pIs) from 6.57 (AcGRF5) to 9.63 (AcGRF4). All AcGRF proteins had a negative hydrophilicity score (GRAVY), indicating that they are hydrophilic proteins. Subcellular localization predictions indicated that seven AcGRF proteins were localized to the nucleus and AcGRF4 was localized to the cell membrane.

**Table 1:** Characteristics of AcGRFs.

Gene name	Gene ID	Chr location	CDS (bp)	Exon no.	Protein length (aa)	MW (kDa)	pI	GRAVY score	Subcellular localization
AcGRF1	Aco009479	LG01:1916306-1917558	903	2	300	33.4	8.18	−0.519	Nucleus
AcGRF2	Aco023268	LG02:11260633-11262425	1,167	4	388	43.3	7.74	−0.887	Nucleus
AcGRF3	Aco020046	LG03:9349100-9351207	927	3	308	34.4	9.00	−0.530	Nucleus
AcGRF4	Aco015543	LG03:12472433-12473582	750	3	249	25.1	9.63	−0.077	Cell membrane
AcGRF5	Aco015755	LG09:10903631-10910491	831	4	276	30.8	6.57	−0.852	Nucleus
AcGRF6	Aco000277	LG12:3038602-3042504	1,839	4	612	64.7	8.03	−0.502	Nucleus
AcGRF7	Aco013343	LG15:11084401-11088716	1,821	4	606	65.7	9.16	−0.711	Nucleus
AcGRF8	Aco013172	LG24:222856-228237	1,200	5	399	43.9	8.80	−0.539	Nucleus

### 3.2 Characterization of AcGRF sequences

**Figure 1:**
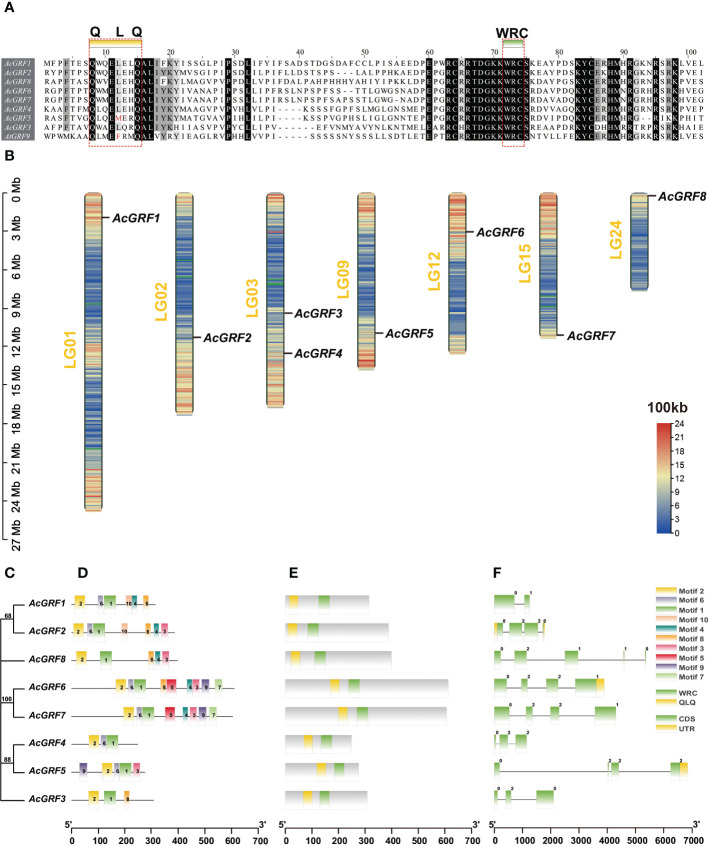
Conserved sequences, chromosomal distribution, gene structures, and motif patterns of *AcGRF* genes and their encoded proteins. **(A)** Conserved sequences in AcGRF genes. The QLQ and WRC domains are marked with a red rectangular box, and the amino acids replaced in the QLQ domain are indicated in a red font. **(B)** Chromosomal distribution of *AcGRF* genes. The sliding window size was set to 100 kb, and the color from red to blue indicates high to low gene density. Blank areas on chromosomes are genetic regions for which information on gene distribution is lacking.**(C)** Phylogenetic clustering of AcGRF proteins. **(D)** Motif patterns of AcGRF proteins. **(E)** Conserved domains in AcGRF proteins. **(F)** *AcGRF* gene structures. Yellow boxes indicate 5′ and 3′ UTR regions; green boxes indicate exons; black lines represent introns; and the number (0–2) indicates the intron phase.

All the pineapple GRFs contained a highly conserved WRC protein domain with the RTDGKKWRC motif. Seven contained the canonical QLQ (Gln-Leu-Gln) motif; the exception was AcGRF5, in which Leu was replaced by Met (Gln-Met-Gln) (**Figure 1A**). A similar result was reported in *A. thaliana*, in which the Leu in AtGRF9 is replaced by Phe (Kim et al., 2003).

The scattered distribution of *AcGRF* gene members on different chromosomes is shown in **Figure 1B**. There are two *GRF* genes on chromosome 3, but they are physically distant. Most *AcGRF* genes tend to cluster in regions with higher gene density.

A conserved motif search was performed on the eight AcGRF proteins, and the number of conserved motifs in individual proteins ranged from 3 to 9. Motifs 1 and 2 were components of the WRC and QLQ domains, respectively, and were present in all GRF proteins, whereas other motifs were only present in certain members. AcGRF proteins in the same subgroup had similar motif compositions (**Figures 1C–E**, **S1**). Exon numbers ranged from 2 to 5, and most *GRF* genes did not have 5′ or 3′ UTR annotation information (**Figure 1F**). Four was the most common number of exons (4 *AcGRF* genes), followed by three (2 genes).

### 3.3 Phylogenetic relationships of the AcGRFs

**Figure 2:**
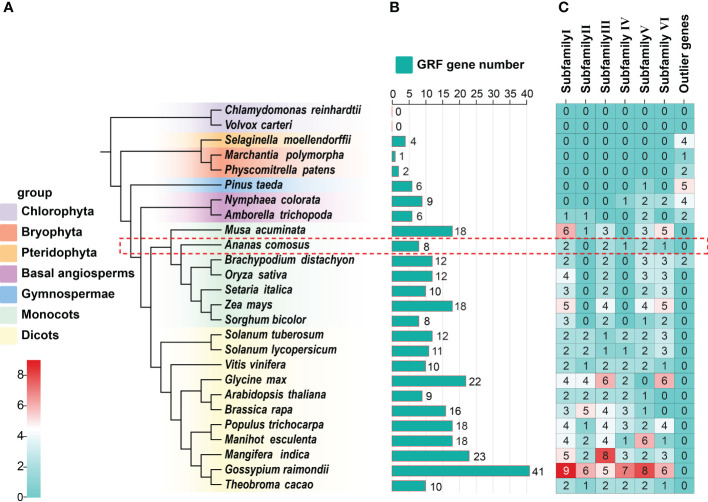
Evolutionary relationships among 26 species and their GRF gene compositions. **(A)** Phylogenetic relationships among 26 species. **(B)** Numbers of GRF genes in different species. **(C)** Distribution of GRF genes in various subfamilies. Phylogenetic analyses of 26 species were performed using OrthoFinder (Emms and Kelly, 2019). The *GRF* genes in pineapple are marked with a red rectangular box.

To reveal the evolutionary relationships among GRF genes from various species, we constructed a phylogenetic tree of *GRFs* from 26 species (**Figure 2A**). GRF genes were identified from 23 additional plant species and two algal species using the same method applied to pineapples (**Figures 2B, C**). Most species had around 10 GRF genes; exceptions included *Marchantia polymorpha* and *Physcomitrella patens* (1), *Musa acuminata* (19), and *Gossypium raimondii* (41) (**Figure 2B**). We constructed phylogenetic trees of the GRF protein sequences from these species using the maximum likelihood method, dividing them into specific subfamilies (**Figures 2C**, **S2**; **Table S1**). Pineapples contained members of all subfamilies except subfamily II: *AcGRF6/7* in subfamily I, *AcGRF1/2* in subfamily III, *AcGRF4/5* in subfamily V, and *AcGRF3* and *AcGRF8* in subfamilies IV and VI, respectively (**Figure S2**). Across all species, subfamily I had the most members, followed by subfamilies III, VI, and V (**Figure 2C**), which accounted for 21.38%, 18.42%, 17.10%, and 16.78% of all GRF genes in the 26 species, respectively. Interestingly, GRF genes from bryophytes and ferns were not grouped into any of the six subfamilies but were present in a separate outer group. *GRFs* from basal angiosperms (i.e., *N. colorata* and *A. trichopoda*) were found in three of the six subfamilies, and some were in the outer group. Banana (no subfamily IV) and pineapple (no subfamily II) contained members of five *GRF* subfamilies, and other monocots contained members of only four subfamilies (no subfamilies II or IV). All dicots contained members of all six subfamilies, with the exception of soybean, which lacked members of subfamily V.

### 3.4 Origins of AcGRF gene members

**Figure 3:**
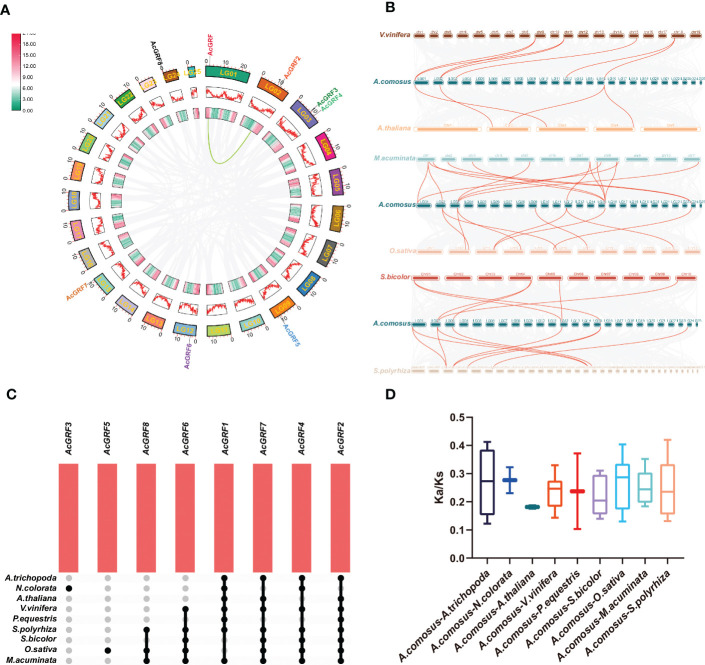
Collinearity and Ka/Ks analyses of pineapple *AcGRFs* and their homologs in other species. **(A)** Circos plot of pineapple collinear homologous genes. Genome-wide collinear blocks are set against a gray background, and duplicate AcGRF gene pairs are highlighted by red and green curves. Each pineapple chromosome is accompanied by 100 kb of gene density information, depicted by heat maps and waveform maps. **(B)** Collinearity of GRF genes in pineapple and six representative species. Same-line blocks are set to a gray background, and collinear GRF genes are highlighted with red curves. **(C)** Venn diagram of non-redundant collinear GRF genes in pineapple and other species. **(D)** Box plot of Ka/Ks ratios of *GRF* orthologs.

Gene duplication is thought to be the main driver of species evolution and a direct cause of gene family expansion (Lynch and Conery, 2000; Moore and Purugganan, 2003; Maere et al., 2005). Pineapple experienced two whole-genome duplication (WGD) events in its early evolution, corresponding to two peaks in **Figure S3**. We searched for tandem and segmental duplicates among the *AcGRFs* (**Tables S2**, **S3**) and visualized them using the Circos plot (**Figure 3A**). Expansion of the AcGRF gene family appeared to have occurred mainly through segmental duplication, and no tandem duplication events were found. There was one segmentally duplicated gene pair (**Figure 3A**); the AcGRF1–AcGRF2 gene pair was associated with the WGD events of pineapple.

To understand the ancestral relationships among AcGRF genes and GRF genes from other species, we examined collinear relationships among genes from pineapple and two core eudicots (*A. thaliana*, *V. vinifera*), two basal angiosperms (*A. trichopoda*, *N. colorata*), and five core monocots (*O. sativa*, *S. bicolor*, *P. equestris*, *M. acuminata*, and *S. polyrhiza*). Four GRF genes from *A. thaliana* had collinear relationships with AcGRF genes from pineapple; the numbers of collinear genes in other species were 7 in *V. vinifera*, 14 in *M. acuminata*, 9 in *O. sativa*, 6 in *S. bicolor*, 9 in *S. polyrhiza*, 2 in *P. equestris*, and 3 in *N. colorata* (**Table S4**; **Figures 3B, C**). *AcGRF2* had collinear relationships with gene(s) in all species except *N. colorata*; the collinear block in which *AcGRF1* is located is present in species other than rice (*O. sativa*) and *S. bicolor*, and the collinear block in which *AcGRF4* is located is present in species except *A. thaliana* and *P. equestris*. Interestingly, *AcGRF5*-related collinear blocks were found only in *O. sativa*, and *AcGRF3*-related blocks were found only in water lilies (*N. colorata*). We next calculated the Ka/Ks (non-synonymous/synonymous substitution ratio) values of *AcGRF* genes and their orthologs in nine other species (**Tables S4**, **S5**; **Figure 3D**). All AcGRF orthologous gene pairs had Ka/Ks values less than 1, indicating that the AcGRF gene family has experienced strong purifying selection during evolution.

### 3.5 Promoter analysis and expression of AcGRF genes

**Figure 4:**
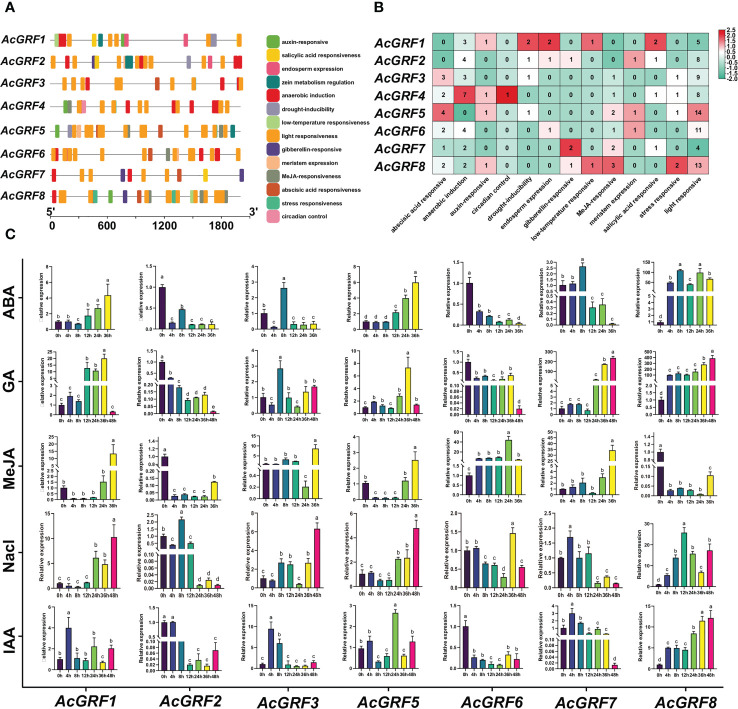
*cis*-Elements in AcGRF gene promoters and AcGRF expression profiles under hormone and salt stress treatments. **(A)** *cis*-Acting elements in the 2,000-bp region upstream of the AcGRF genes. **(B)** Distribution of cis-Acting elements of the AcGRF genes. **(C)** AcGRF gene expression profiles under different hormone and salt stress treatments. Data were normalized to the expression of β-actin and expressed as mean ± standard deviation. Different lowercase letters indicate significant differences between treatment times (*P* < 0.01, F test).

Gene promoters interact with DNA or regulatory proteins to control gene expression (Biłas et al., 2016). We extracted promoter sequences from the pineapple GRF genes (2,000 bp upstream of the start codon) and identified their *cis*-acting elements. A total of 156 *cis*-elements were detected in the promoter regions of the AcGRF genes and were divided into three classes (**Figures 4A, B** and **Table S6**). Hormone response elements included those responsive to IAA (4; 2.56%), GA (4; 2.56%), ABA (14; 8.97%), JA (10; 6.41%), and salicylic acid (5; 3.21%). With the exception of subfamily III members (*AcGRF1/2*), the promoter regions of all other subfamily members contained JA and ABA response elements. Growth- and development-related elements included those related to meristem expression (3; 1.92%; present in *AcGRF1* and *AcGRF8*), endosperm expression (4; 2.56%; present in *AcGRF1/2/7*), light response (72; 46.15%; present in all AcGRF genes), and circadian rhythm control (1; 0.64%; present in *AcGRF4*). Stress response elements included those related to drought induction (5; 3.21%), low temperature response (2; 1.28%), and anaerobic induction (25; 16.03%). Anaerobic induction elements were present in all genes except *AcGRF5*, and other response elements were unevenly distributed among the *AcGRFs*.

Studies have reported that GRF genes have a positive regulatory effect on callus proliferation (Kong et al., 2020), and GRF genes regulate plant growth and development by regulating GA, IAA, and ABA (Kim et al., 2012; Chen Y. et al., 2020; Huang et al., 2022). To investigate whether the expression of AcGRF genes is affected by abiotic stress and hormone treatments, we measured the expression of seven *AcGRF* family members by qRT-PCR after exposure to different hormones or 150 mM NaCl (**Figure 4C**). *AcGRF1* expression was induced by all tested treatments. The expression of *AcGRF1* and *AcGRF5* gradually increased with ABA exposure time, whereas that of *AcGRF6* showed the opposite trend. GA significantly induced *AcGRF1*, *AcGRF7*, and *AcGRF8* expression and inhibited that of *AcGRF2* and *AcGRF6*. JA significantly induced the expression of *AcGRF6*. IAA significantly induced the expression of *AcGRF8* but inhibited that of *AcGRF6*. Most *AcGRF* genes increased in expression with increasing duration of NaCl exposure. *AcGRF8* had the highest expression levels during ABA and GA treatment, with relative expression levels of 110 and 387, respectively, and *AcGRF7* had a relative expression level of 237 after GA treatment. These findings suggest potential roles for AcGRF genes under different hormone treatments and stress conditions.

**Figure 5:**
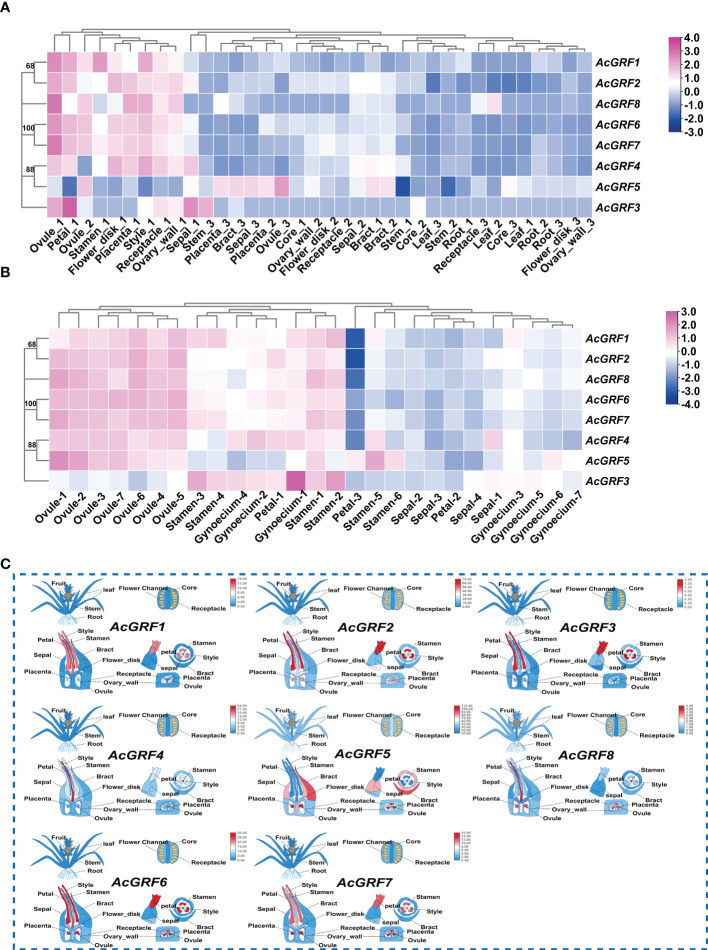
Expression of AcGRF genes in different tissues of pineapple. **(A)** Clustered heat map showing the expression patterns of AcGRF genes in various tissues at different developmental stages. **(B)** Expression heat map of AcGRF genes in five floral organs (stamens, pistils, ovules, sepals, and petals) of pineapple at different developmental stages: four stages of sepals (S1–S4), three of petals (S1–S3), five of stamens (S1–S5), and seven of pistils (S1–S7) and ovules (S1–S7). Relative gene expression is quantified as log_2_(FPKM+1). Blue, white, and red indicate low, medium, and high expression levels. **(C)** Cartoon heatmap showing the expression patterns of AcGRF genes from different clades in different tissues.

We next analyzed the expression profiles of GRF genes at different developmental stages of pineapple tissues using our previously reported transcriptome data (Mao et al., 2018) (**Figure 5** and **Table S7**). The analysis included roots, stems, leaves, petals, stamens, pistils, discs, peduncles, ovules, ovaries, fruit hearts, bracts, sepals, and placenta (**Figure 5A**). In general, GRF genes from the same clade tended to exhibit similar expression patterns. Subfamily I members *AcGRF6* and *AcGRF7* showed a similar expression in nearly all tissues tested. Interestingly, almost all AcGRF genes were relatively highly expressed in the early stages of floral organ development (**Figure 5A**), with the exception of *AcGRF5*, which was relatively highly expressed in the late developmental stages of most tissues.

To determine whether individual AcGRF genes are associated with specific developmental stages of pineapple floral organs, we analyzed transcriptome data from five floral organs (sepals, petals, stamens, pistils, and ovules) at different developmental stages (Wang et al., 2020). Almost all AcGRF genes showed the highest expression in ovules at all stages of development, followed by stamens; the only exception was *AcGRF3*, which was specifically expressed in pistils (**Figure 5B** and **Table S8**).

To better visualize the different expression patterns of AcGRF genes in various tissues, we created cartoon heatmaps of relevant gene expression from all pineapple *GRFs* (**Figure 5C**). Subfamily III member *AcGRF1* was highly expressed in petals, stamens, and pistils. *AcGRF2* was highly expressed in petals and ovules, and subfamily VI member *AcGRF8* was expressed at higher levels in pistils and ovules. Subfamily I members *AcGRF6* and *AcGRF7* were highly expressed in petals, pistils, and ovules. *AcGRF4* from subfamily V was highly expressed in pistils, and *AcGRF5* was highly expressed in bracts, sepals, and ovules. Subfamily IV member *AcGRF3* showed relatively high expression in petals. The expression patterns of AcGRF genes thus differ among pineapple tissues, suggesting their potential regulatory roles in pineapple development.

### 3.6 Predicted protein interaction network of AcGRFs

**Figure 6:**
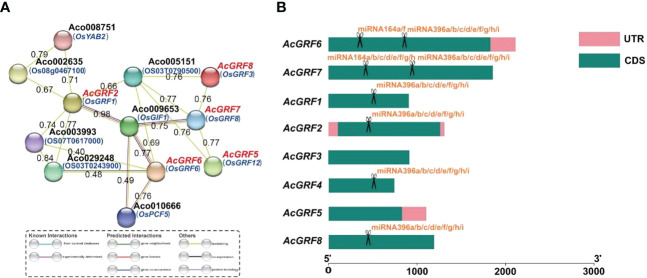
Predicted protein interaction network of AcGRF proteins and miRNA target sites in *AcGRF* genes. **(A)** Protein interaction network predicted using *AcGRF* orthologs from rice. **(B)** Predicted miRNA targets in the *AcGRF* genes. The scissors represent the miRNA and its targeted *AcGRF* gene, with penalty score ≤5 and lower expectations indicating higher prediction accuracy.

A predicted protein interaction network indicated that AcGRFs had multiple interaction partners (**Figure 6A**), including growth regulator interaction factor 1 (GIF1), the TCP (teosinte branched1/cincinnata/proliferating cell factor) transcription factor PCF5, and the YABBY transcription factor YAB2. With the exception of AcGRF1/3/4, all other members interacted with one or more proteins. AcGRF2/5/6/7/8 were predicted to interact with 6, 2, 5, 4, and 2 proteins, respectively. There were also predicted interactions between the AcGRF proteins themselves.

We next performed miRNA target site prediction for the *AcGRF* genes. As shown in **Figure 6B**, six *AcGRF* genes were predicted to be targeted by two miRNAs (miRNA164/miRNA396). *AcGRF6/7* contained targets for both miRNAs, whereas *AcGRF1/2/4/8* contained targets for miRNA396 only (**Table S9**). Interestingly, all miRNA target sites were located in the CDS regions.

## 4 Discussion

GRF proteins regulate important processes in plant biology, including leaf growth, floral organ development, root development, seed oil content, plant lifespan, and stress response (Omidbakhshfard et al., 2015). Pineapple is the third most important tropical fruit crop in the world after bananas and citrus, and various biotic and abiotic stresses have a significant impact on its yield and quality. Although GRF genes have been studied in other plant species, they have not yet been characterized in the bromeliad family. We therefore identified the GRF genes in pineapple, analyzed their evolutionary relationships, compared their sequence features, and analyzed their expression patterns in response to hormone and salt stress treatments.

### 4.1 Identification, classification, and characteristics of the *AcGRF* genes

We identified eight AcGRF proteins from the pineapple genome and assigned them to five subfamilies on the basis of phylogenetic analysis (**Figures 2A, C**). Pineapple has fewer GRF genes than the monocots banana (18) and maize (18) but a similar number to *Setaria italica* and *S. bicolor*. This suggests that *GRF* family expansion in monocots may be associated mainly with lineage-specific WGD events. Pineapple contained members of five of the six major *GRF* subfamilies, similar to rice (four major subfamilies), banana (5), *B. distachyon* (4), *A. thaliana* (5), soybean (5), and *B. rapa* (5) and more than non-angiosperm species such as *M. polymorpha* (0), *S. moellendorffii* (0), and *Pinus taeda* (1). Notably, genes that fell outside the main subfamilies were found only in algae, ferns, gymnosperms, basal angiosperms, and *Brachypodium distachyon*; they may have been preserved from more ancient duplication events or acquired only in specific clades. Among monocots, there were five *GRF* subfamilies in banana (missing subfamily IV) and pineapple (missing subfamily II) and four subfamilies in other species (missing both subfamily II and subfamily IV). By contrast, all dicots except soybean (missing subfamily V) contained members of all six subfamilies. These results suggest that the preservation of specific subfamilies may vary among species with different evolutionary histories. Several genomic evolutionary models have been proposed in model species on the basis of comparative genomic analysis (Wolfe and Shields, 1997; Moore and Purugganan, 2003; Hurley et al., 2005). If duplicated genes are retained, they tend to diverge in their regulatory and coding regions, and differences in coding regions, especially those that alter gene function, may cause amino acid changes or substitutions or changes in exon/intron structure (Xu et al., 2012). In general, proteins from the same family have relatively conserved intron/exon structures, such as β-expansins in *A. thaliana* and rice (Lee and Kende, 2001). However, the number and length of introns are not conserved in GRF genes from pineapple, *A. thaliana*, and rice (**Figure 1C**), even within the same subfamily, and GRF genes are randomly distributed in the genome (**Table 1** and **Figure 1B**). This suggests that no recent duplication events have occurred in the GRF gene family and that expansions in this gene family reflect early duplication events.

Gain and loss of exons/introns and differences in exon/intron length can result from chromosome rearrangement and fusion (Zan et al., 2020). In pineapple, *AcGRF2* contains three introns, whereas its paralog *AcGRF1* contains only one intron. Likewise, intron lengths were much greater in *AcGRF5* than in its paraphyletic homologous gene *AcGRF4*. We therefore speculate that differences in intron/exon length or number may have led to different biological functions for *AcGRF4* and *AcGRF5*. Previous studies have reported that the N-terminal QLQ domain is involved in protein–protein interactions (Kim et al., 2003; Kim and Kende, 2004). Here, we found that the QLQ motif of AcGRF5 is actually Gln-Met-Gln, with a Met substituted for Leu (**Figure 1A**), similar to that of *A. thaliana* AtGRF9 in which Phe is substituted for Leu (Kim et al., 2003). The mutated QLQ domain of AcGRF5 may lead to alterations in its protein interaction activity.

### 4.2 Evolution of the AcGRF genes

Gene duplication, including tandem, segmental, and whole-genome duplication, is one of the main drivers of genome evolution (Moore and Purugganan, 2003), and most seed plants have experienced one or more WGD events in their evolutionary history (Rensing et al., 2007). Pineapple has undergone at least two WGD events (Ming et al., 2015; Ming et al., 2016) (**Figure S3**), and its gene family expansion reflects the effects of WGDs, as well as tandem and segmental duplications. In this study, we analyzed the presence of GRF genes in 26 species, including algae, mosses, angiosperms, and gymnosperms (**Figures 2B, C**). GRF genes were present in all species except algae, suggesting that GRF genes appeared in land plants and may have supported their terrestrial adaptation. Two of the eight pineapple GRF genes were associated with segmental duplication events (**Figure 3A**), consistent with findings in wheat that 26 of 30 GRF genes were associated with such events (Zan et al., 2020). This suggests that segmental duplication may have played an important role in the early expansion of the GRF gene family. The number of *AcGRF* genes was significantly lower in pineapple than in banana (18) compared with other plants used for collinearity analysis, and it was similar to the number of GRF genes in other plant species (Osnato et al., 2010; Kim et al., 2012; Debernardi et al., 2014; Kuijt et al., 2014; Omidbakhshfard et al., 2015). The large number of banana *GRFs* may thus reflect banana-specific duplication events. Notably, there was no significant correlation between the number of GRF genes and genome size. For example, *A. thaliana* has nine *GRFs* and pineapple has eight, despite the pineapple genome being 3.2 times the size (375 Mb) (Ming et al., 2015) of the *A. thaliana* genome (115 Mb) (Hou et al., 2022). After identifying non-redundant GRF genes that showed collinear relationships between pineapple and nine other species, we found that an *AcGRF5* ortholog was present only in pineapple and rice and an *AcGRF3* ortholog was present only in pineapple and water lily (**Figure 3C**). This suggests that the retention of gene family members can vary among species with different evolutionary histories. All *AcGRF* orthologous genes had Ka/Ks values less than 1 (**Figure 3D**), indicating that *GRFs* experienced strong purifying selection during evolution.

### 4.3 Inference of biological functions of *AcGRF* genes

Because of the importance of pineapple as a tropical fruit crop and ornamental plant, the mechanisms that regulate its flower and fruit development are of significant interest. The biological functions of pineapple AcGRF genes remain to be clarified, but identification of their presumed orthologs in different species can provide insight into their functions (Nehrt et al., 2011). GRF genes have previously been studied in other species to characterize their regulatory network and understand their functions in a wide range of biological processes (Omidbakhshfard et al., 2015). Here, we used GRFs with known functions in other species to infer potential functions of their pineapple orthologs (**Figure S4** and **Figure 6**). Clade 2 includes *AcGRF1/2*, *AtGRF6*, and *OsGRF1*. Previous studies have shown that *AtGRF6* and *OsGRF1* have a role in controlling leaf size (Kim et al., 2003; Luo et al., 2005) and *OsGRF1* also influences rice stem elongation, juvenile growth, and panicle extension (Van der Knaap et al., 2000; Luo et al., 2005). *AcGRF1/2* in the same clade were highly expressed in early developmental stages of petals and ovules, suggesting that they may have similar functions in pineapple. *ZaGRF6*, *OsGRF3*, and *AcGRF8* were included in clade 3. Heterologous overexpression of *ZaGRF6* increased branching and chlorophyll synthesis and delayed aging in transgenic tobacco, and ectopic overexpression of *OsGRF3* in rice reduced tiller numbers and induced the formation of ectopic roots and shoots on the nodes (Kuijt et al., 2014; Zhang et al., 2021). Promoter analysis revealed the presence of two ABA response elements and one gibberellin response element in the *AcGRF8* gene promoter, and qRT-PCR results showed that *AcGRF8* expression responded significantly to ABA and GA treatment. These results suggest that *AcGRF8* may regulate pineapple aging and root and shoot formation through the abscisic acid and gibberellin pathways. Clade 5 included *AcGRF4/5*, *AtGRF9*, *OsGRF10*, and *ZmGRF10*. In previous studies, overexpression of *ZmGRF10* in maize led to reductions in leaf size and plant height, and knockout and overexpression of *AtGRF9* made petals and other organs of *A. thaliana* larger and smaller, respectively (Wu et al., 2014; Omidbakhshfard et al., 2018). Here, *AcGRF4* was preferentially expressed in pistils and *AcGRF5* in bracts, sepals, and ovules. *AcGRF4* and *AcGRF5* have IAA response elements, and their expression responded to increasing durations of IAA treatment. We therefore suggest that *AcGR4/5* may participate in the regulation of floral organ and leaf size, plant height, and other traits through the auxin pathway. Clade 6 included *AcGRF3*, *AtGRF7*, and *AtGRF8*. Leaves of an *AtGRF7* single-allele mutant were reported to be smaller than those of the wild type (Kim et al., 2012), but this phenomenon was not observed by Lee et al. (2022). *AtGRF8* has been reported to positively regulate cell proliferation (Tsukaya, 2021). We found that *AcGRF3* was preferentially expressed in petals and contained GA-responsive elements in its promoter. We therefore speculate that *AcGRF3* may regulate pineapple cell proliferation, leaf size, and petal growth through the GA pathway. Clade 8 included *AcGRF6/7*, *PagGRF15*, and *OsGRF7/8*. *PagGRF15* regulates leaf size through its effects on cell expansion during poplar leaf development, and *OsGRF7/8* influence plant structure and leaf growth by regulating gibberellin and indole-3-acetic acid metabolism. Here, *AcGRF6* and *AcGRF7* were preferentially expressed in petals, pistils, and ovules. qRT-PCR experiments showed that GA significantly induced the expression of *AcGRF7* but inhibited that of *AcGRF6*, and IAA significantly inhibited *AcGRF6*. *AcGRF6/7* may therefore regulate pineapple flower and leaf development and plant structure through GA and IAA pathways.

## 5 Conclusion

We identified eight GRF gene family members on seven chromosomes of the pineapple genome and classified them into five of six known subfamilies on the basis of phylogenetic analysis. In contrast to other gene families, the GRFs showed little conservation of gene structure and motif composition. Collinearity analysis showed that early segmental duplications promoted expansion of the pineapple GRF gene family, and purifying selection was the main force acting on the GRF genes. The paralogs *AcGRF1* and *AcGRF2* had different expression profiles in different floral organs, perhaps related to their differences in structure. Transcriptome data suggested that all AcGRF genes were involved in regulation of floral organ development, and *AcGRF1/2/3/6/7* may have functionally redundant roles in petal development. The *AcGRF* promoters contained *cis*-acting elements involved in hormone response, developmental regulation, and stress response, and changes in expression of *AcGRFs* under ABA, GA, IAA, JA, and NaCl treatments may reflect their different regulatory effects. Protein interactions between AcGRF proteins or with YABBY, TCP, and AcGRF-miRNA may contribute to the formation of more complex regulatory networks. These results support further research on the functions and regulatory mechanisms of AcGRF transcription factors during pineapple growth and development.

## Data availability statement

The datasets presented in this study can be found in online repositories. The names of the repository/repositories and accession number(s) can be found in the article/**Supplementary Material**.

## Author contributions

YH, CC conceived the project. WY and JW performed the experiments. WY, AL, JW, CL and WZ performed data analysis. WY and AL wrote the original manuscript draft. All authors participated in discussion and revised the manuscript. All authors reviewed and approved the manuscript.
