# Supplementary figures and images for: Genome-wide identification, phylogeny, and expression analysis of GRF transcription factors in pineapple (Ananas comosus)
Source: Front Plant Sci. 2023 Apr 14;14:1159223. doi: 10.3389/fpls.2023.1159223 (PMC10140365; doi:10.3389/fpls.2023.1159223)

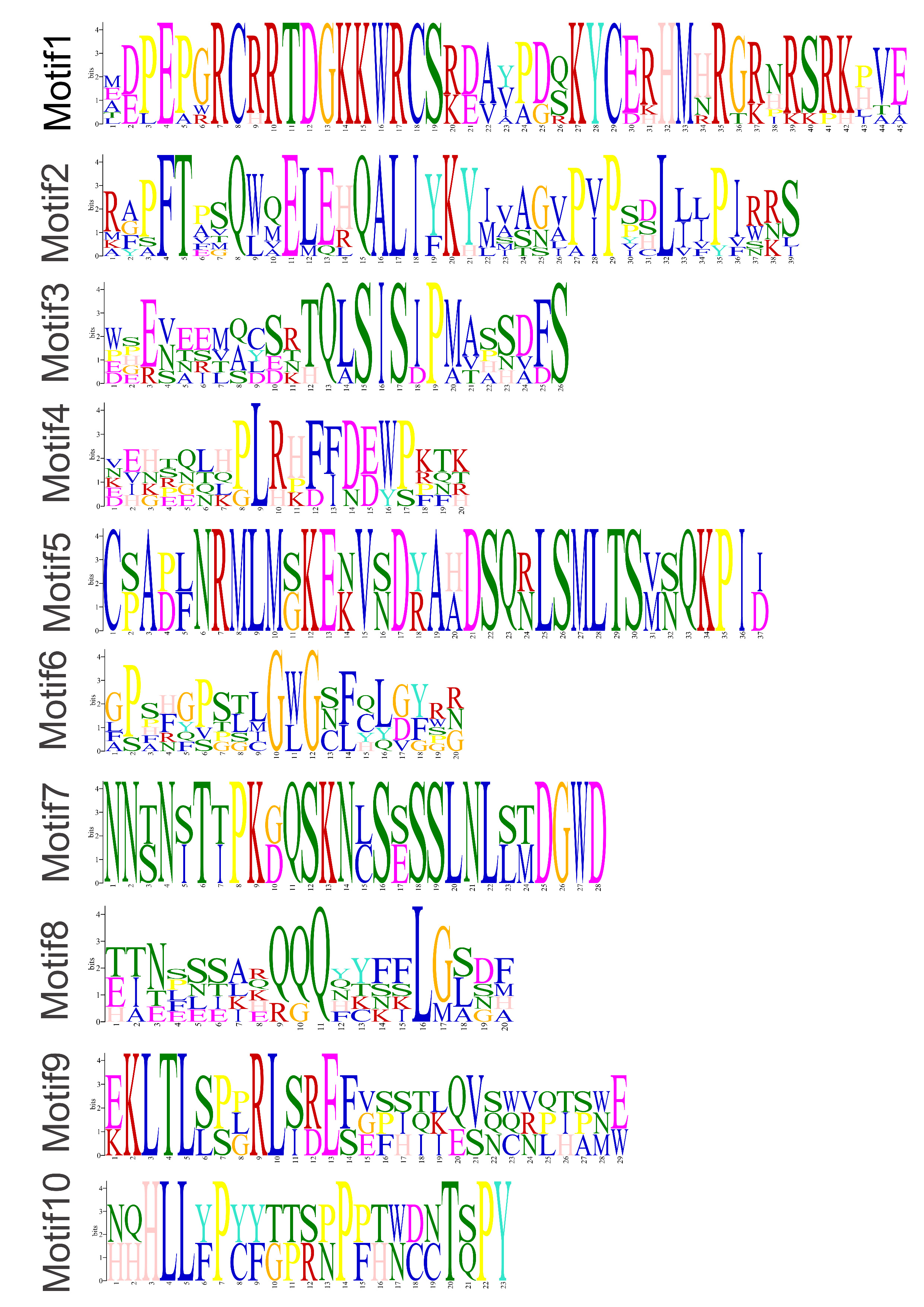

Supplement: Supplementary file 1 [file Image_1.jpg]

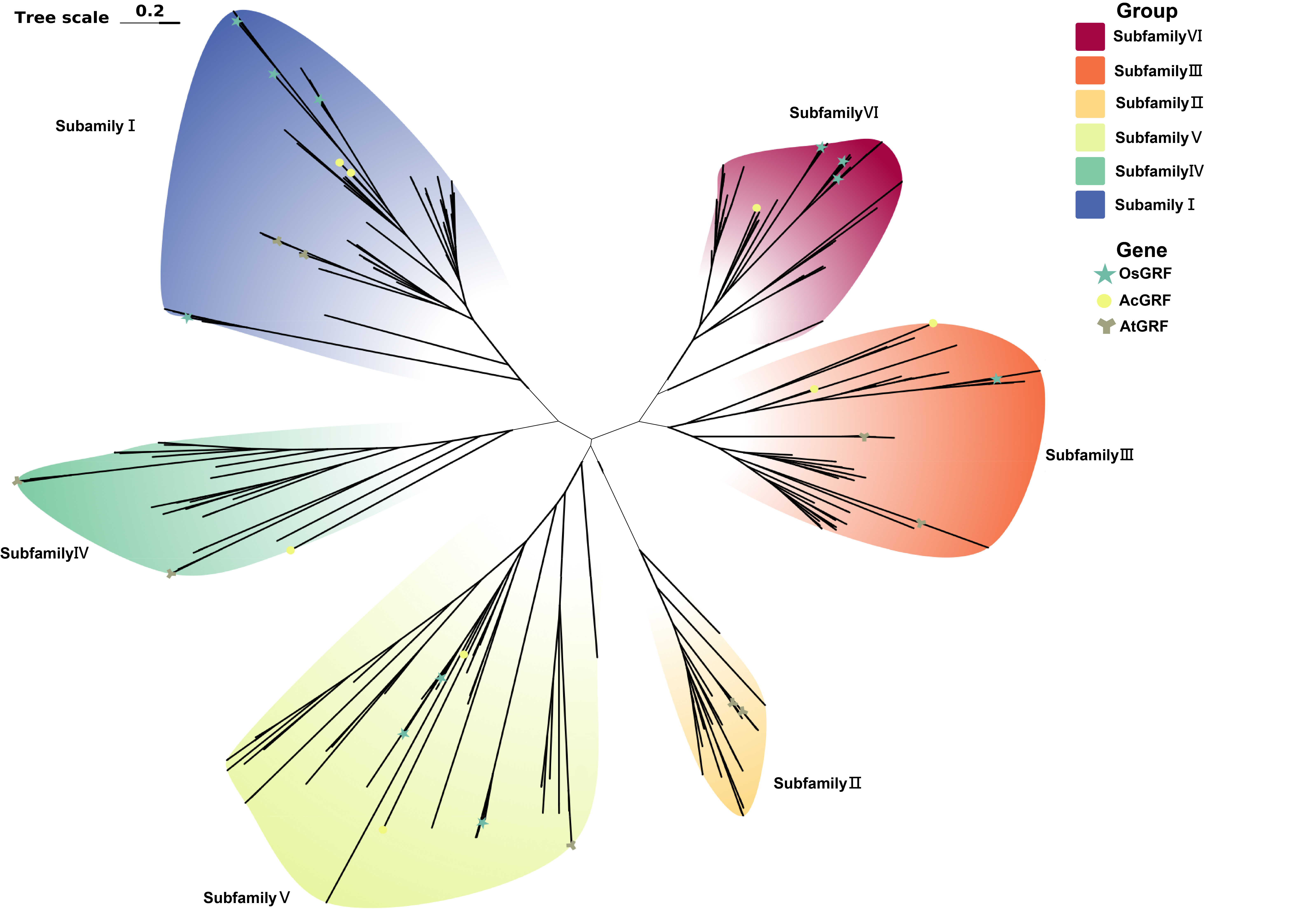

Supplement: Supplementary file 2 [file Image_2.jpg]

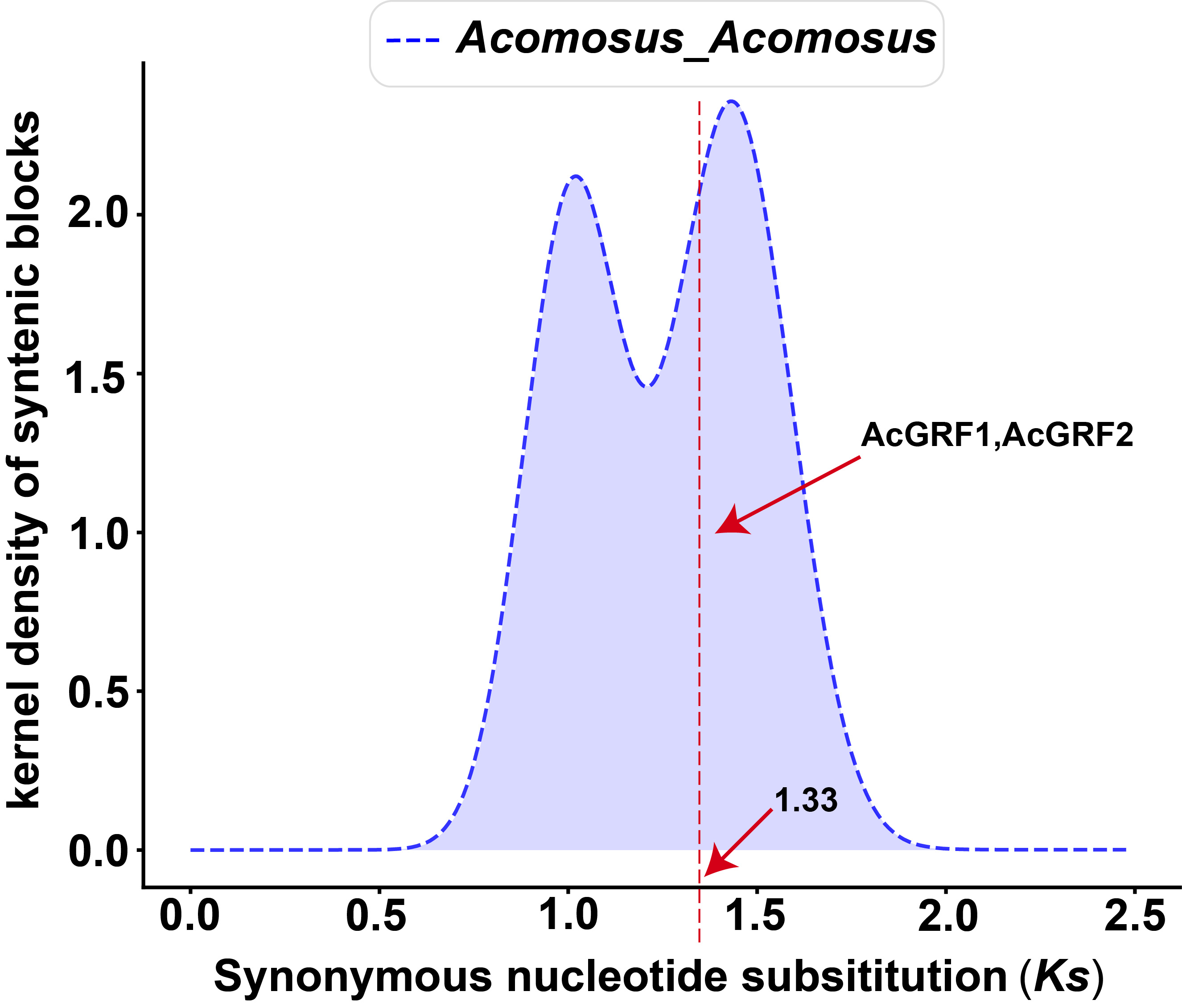

Supplement: Supplementary file 3 [file Image_3.jpg]

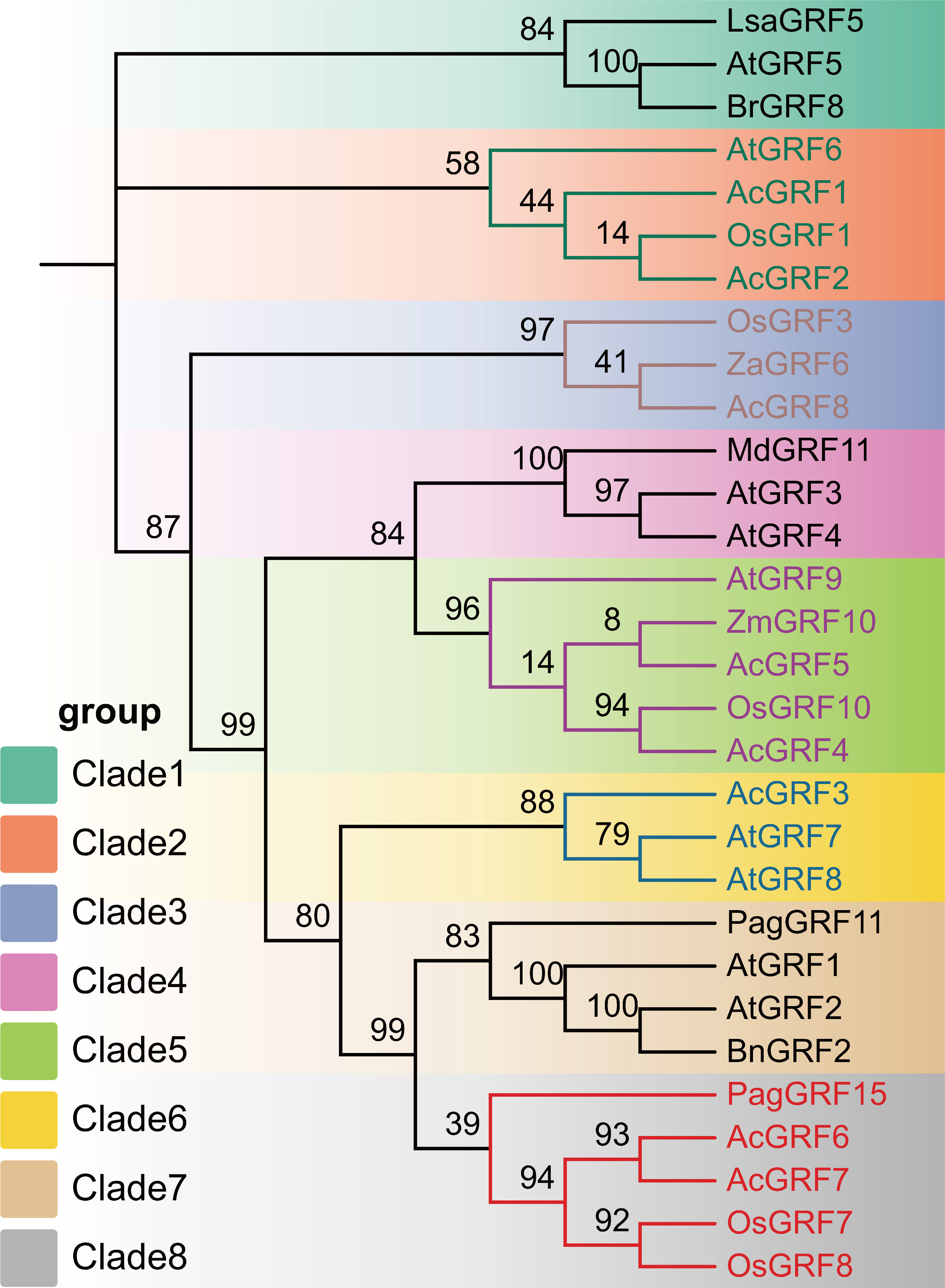

Supplement: Supplementary file 4 [file Image_4.jpg]
